# Knowledge of gym goers on myths and truths in resistance training

**DOI:** 10.1038/s41598-025-87485-8

**Published:** 2025-01-27

**Authors:** Alexandra Unger, Clemens Mosgan, Christofer Wolte, Sara Pettauer, Jan Wilke

**Affiliations:** 1 Department of Movement Science, Institute of Sports Science, University of Klagenfurt, Klagenfurt, Austria; 2 Department of Neuromotorics and Movement, Institute of Sports Science, University of Bayreuth, Bayreuth, Germany; 3 University of Teacher Education Carinthia, Klagenfurt, Austria

**Keywords:** Strength, Nutrition, Hypertrophy, Implementation, Health care, Medical research

## Abstract

Over the last decades, resistance training (RT) has experienced a surge in popularity, and compelling evidence underpins its beneficial effects on health, well-being, and performance. However, sports and exercise research findings may translate poorly into practice. This study investigated the knowledge of Austrian gym-goers regarding common myths and truths in RT. Based on topical systematic reviews (n = 45), a digital questionnaire with 14 statements on RT methods and practices, randomly phrased as myths or truths, was administered to Austrians exercising in local fitness centres. Chi-squared tests were used to check if a majority correctly identified true and false statements. A total of n = 721 (30.1 ± 14 years, 454 males) out of n = 790 contacted individuals completed the survey (response rate: 91.2 percent). Five out of fourteen statements (truth: protein augments strength and hypertrophy, creatine augments strength, superiority of full- vs. partial-ROM RT for hypertrophy; myth: RT reduces flexibility, low-load, high-volume RT is as effective as high-load RT with regard to maximal strength) were identified correctly by a majority (p < .01). The awareness of the scientific evidence on RT is low among gym-goers. Upcoming studies should be geared to improve science communication.

## Introduction

The World Health Organization recommends adults to engage in muscle-strengthening activities at least twice per week to sustain or enhance overall health. Notably, resistance training (RT) is particularly effective in promoting muscle hypertrophy compared to both sedentary lifestyles and aerobic exercise^1^. Given that muscle mass declines by 3 to 8% each decade after age 30 (approximately 0.2 kg of lean weight loss per year) and by 5 to 10% each decade after age 50 (approximately 0.4 kg of lean weight loss per year), the importance of regular RT becomes even more evident^2,3^. A wealth of evidence confirms the beneficial effects of RT on health outcomes^4–7^. For instance, RT improves cardiometabolic health by lowering blood lipids, blood pressure, as well as the risk of diabetes type II and coronary heart disease^8–10^. RT, furthermore, reduces the risk of falls^11^, alleviates orthopaedic complaints such as low back pain^12^ or osteoarthritis^13^, and represents a pivotal component of exercise programs in the prevention and rehabilitation of sports injuries^7,14,15^. The health effects of RT also include mental aspects as RT decreases the symptoms of depression and anxiety while improving self-esteem, self-concept, and cognitive function^7,16–18^. From a global perspective, regular performance of RT reduces all-cause mortality in healthy adults but also in diseased populations such as cancer survivors^19–22^.

In view of the manifold benefits of muscle-strengthening, the last decades have been characterized by a surge in the popularity of RT. In Europe, the membership rates of commercial fitness centres have grown about 4% per year, actually holding a total rate of about 10%. This means that almost each fifth person is registered in a gym^23^. However, researchers have claimed that several factors, particularly the perceived complexity of RT and the need for specialized equipment and knowledge, prevent a higher participation^24^. Indeed, RT allows for considerable variation with regard to the tools used (e.g. machines, body weight, resistance bands, free weights) and training-related variables (e.g., type and order of exercises, weight/intensity, number of sets and repetitions, training frequency and rest duration). Similarly, RT can have numerous objectives, including the improvement of motor function (e.g., strength, power, local muscle endurance, speed, agility, balance, coordination, explosiveness) as well as the modification of morphology and body composition (e.g., weight loss, muscle hypertrophy, body shaping^25,26^. In addition, several nutritional options exist to optimize RT through both modification of dietary sources and supplementation (e.g., protein, carbohydrates, creatine).

The members of fitness centres are frequently on their own after a general familiarization with devices and exercises and may train far from scientifically based training foundations. Interestingly, research has shown that not even health professionals, experts or trainers are substantially aware of the large body of training knowledge accrued over the last decades^27,28^. For instance, a recent study showed that the surveyed physiotherapists and sports scientists misclassified myths and truths about the effects of stretching in more than half of the cases^29^. As RT is a similarly popular and well-researched topic, it is of interest as to whether end users are aware of the validity of the various recommendations in the field of RT (e.g. optimal intensity/volume or protein supplementation). The present study, therefore, aimed to investigate the knowledge of individuals regularly training in gyms with regard to myths and truths about RT. Considering the widespread popularity, the diverse trainings levels and the different exposures to RT, we hypothesized that there would be a significant discrepancy between the available scientific evidence and the knowledge of gym-goers.

## Methods

### Ethics and study design

The present study included two parts. First, the evidence on methods and strategies used to maximize the effectiveness of RT was analysed evaluating topical systematic reviews. Second, a cross-sectional survey on the awareness about the evidence among gym-goers was performed, recruiting participants in fitness centres of the largest cities (Klagenfurt, Villach, Wolfsberg, Spittal an der Drau and Feldkirchen) in the Austrian state Carinthia. The study adhered to the guidelines for Good Practice in the Conduct and Reporting of Surveys^30^ and was approved by the Ethics Committee of the University of Klagenfurt. All methods were performed in accordance with relevant guidelines and regulations, including the Declaration of Helsinki. Written informed consent was obtained from all participants in the digital survey.

### Evidence synthesis (study part one)

The scope and research questions of the study were derived based on an initial kick-off meeting of the authors who discussed potential myths and truths in resistance exercise. The results of this discussion were supplemented by a systematic review (three independent investigators) of the literature, focusing on training methods, practices, and principles related to RT in healthy adults. Using a combination of structured (including MesH terms and Boolean operators) and free searches, they identified systematic reviews investigating the effects of RT training methods and practices aiming to increase the effectiveness of RT. As an example of the structured searches, we used the following term to obtain relevant papers on protein intake and hypertrophy/strength (all search terms can be found in the supplementary information):

“resistance training” AND protein AND (supplementation OR ingestion OR intake) AND (“muscle mass” OR "lean body mass") AND (“systematic review” OR “Meta analysis”).

The results of the identified reviews were the basis for the construction of the cross-sectional survey administered to gym members in study part two.

### Questionnaire (study part two)

A digital questionnaire capturing gym-goers´ knowledge of myths and truth around RT was generated using a multi-stage expert consensus process. First, all authors evaluated and discussed the results of the literature searches (part one). If topical systematic reviews clearly supported or opposed the use of a strategy or method in RT, it was included as an item of the questionnaire. The phrasing of statements as myths or truths was done randomly in order to avoid positive or negative framing. Face validation was conducted by sending the questionnaire to members of the target population—beginners, intermediate, and experienced members (n = 3 of each group) of a fitness centre. After revising and adapting the questionnaire for wording and comprehension according to the feedback provided, the final instrument consisted of basic demographic questions (age, height, weight, sex, training goal, city of the fitness centre) and specific questions about myths and truths in RT. The included topics were: effect of protein supplementation (hypertrophy/strength, timing of intake, source) and creatine supplementation (strength), impact of carbohydrate intake on RT performance (ability to work out at higher intensity and/or higher training volume), effects of magnesium intake on muscle cramps, relevance of frequency, repetitions and weight (many repetitions at low loads vs. few repetitions at high loads), range of motion (partial/full), equipment (machines/free weights) and sex for muscle hypertrophy and maximal strength. The final instrument comprised a total of 14 single-choice questions (myth/ truth, see supplementary information for the full questionnaire). During data collection, a trained investigator was available to explain terms during the survey if needed.

### Sample (study part two)

In a first step of study part two, we contacted all fitness centres in the five largest cities of the Austrian state Carinthia (Klagenfurt, Villach, Spittal an der Drau, Wolfsberg and Feldkirchen). From the 28 contacted gyms, 20 participated in the study, granting permission to survey their members. Data collection was performed face-to-face between October 2023 and April 2024. Briefly, an investigator directly addressed the participants providing them with a QR code which could be opened on smartphones, tablets, or laptops. Alternatively, they offered reading out the questions and documenting the response for the surveyed individuals. To prevent selection effects due to daytime, each gym was visited in the morning and afternoon/evening. Topical experts (e.g., sports scientists, physiotherapists, public health experts, students of relevant scientific disciplines) were excluded. In total, n = 790 individuals were invited to participate and responses from n = 721 persons (male: 63%, n = 454; female: 37%, n = 267) were obtained which corresponds to a response rate of 91.2%.

### Statistics

The normal distribution of the survey responses was tested by means of the Shapiro–Wilk test. Descriptive statistics (absolute and relative frequencies of correct/incorrect answers) were applied to present the obtained data (means/medians) as appropriate. The χ2 goodness-of-fit test was then used to reveal significant differences between the frequencies of participants choosing the correct answer (e.g. statement is false or true) and those selecting an incorrect answer. Using this procedure, conclusions could be drawn as to whether the majority of the individuals were able to identify myths and truths of RT based on the available scientific evidence. The significance level was set to α = 0.01. Data analyses were performed using JAMOVI, version 2.3 (https://www.jamovi.org).

## Results

### Findings of the literature search

The searches returned a total of 45 systematic reviews covering the 14 topics, with some reviews addressing multiple topics and being relevant to more than one myth or truth. A large quantity of articles (n = 20) focused on RT and supplementation. Eight examined the role of protein supplementation with regard to hypertrophy and strength^31–38^. The timing of protein intake^38–40^ and the source of protein (animal or plant)^41,42^ were investigated by five papers. Three articles focused on the effects of creatine supplementation^43–45^. Two papers each addressed the impact of carbohydrates on RT performance^46,47^ and the relationship between magnesium and cramps^48,49^.

We identified a total of 28 reviews for RT performance and related training practices. Two articles examined the effects of RT on flexibility^50,51^. Seven papers investigated the impact of training load on hypertrophy^52–58^ or maximum strength^52,55,59^, and five reviews focused on the role of training frequency^60–64^. The remaining eleven articles addressed the necessity to perform RT to muscle failure^65–67^, with partial or full ROM^68–71^, with machines or free weights^72,73^, as well as the role of sex^74,75^.

### Sample characteristics

About one half of the gym-goers (54.4%, n = 392) trained in fitness centres in Klagenfurt, the other half were members in the cities of Wolfsberg (12.3%, n = 89), Villach (15.7%, n = 113), Spittal an der Drau (11.5%, n = 83), and Feldkirchen (6.1%, n = 44). The average age of the respondents was 30.1 years (± 14) and a majority were males (63%, n = 454). Almost half of the individuals (44.8%, n = 323) reported hypertrophy as their primary training goal, the rest selected increases of general fitness (32%, n = 231), improvements of strength (9.4%, n = 68), weight loss (7.8%, n = 56), increases of endurance (4.4%, n = 32), and other goals (1.5%, n = 11, Table 1).

**Table 1:** Sample characteristics of the gym-goers.

	Total (n = 721)	Women (37%, n = 267)	Men (63%, n = 454)
Sample characteristics	mean (SD)	mean (SD)	mean (SD)
Age (years)	30.1 (14)	30.4 (13.9)	29.9 (14.1)
Height (cm)	176 (9.25)	167 (6.26)	181 (6.26)
Weight (kg)	75.7 (15.1)	63.3 (9.39)	82 (12.9)
Location of gyms	% (n)	% (n)	% (n)
Klagenfurt	54.4 (392)	20.8 (150)	33.6 (242)
Villach	15.7 (113)	5 (36)	10.7 (77)
Spittal an der Drau	11.5 (83)	4.3 (31)	7.2 (52)
Feldkirchen	6.1 (44)	2.1 (15)	4 (29)
Wolfsberg	12.4 (89)	4.9 (35)	7.5 (54)
Training goal	% (n)	% (n)	% (n)
Hypertrophy	44.8 (323)	12.2 (88)	32.6 (235)
Fitness	32 (231)	15.7 (113)	16.4 (118)
Strength	9.4 (68)	2.8 (20)	6.7 (48)
Weight loss	7.8 (56)	4.6 (33)	3.2 (23)
Endurance	4.4 (32)	1.1 (8)	3.3 (24)
Other settings	1.5 (11)	0.7 (5)	0.8 (6)

*cm* centimetres, *kg* kilogram, *SD* standard deviation.

### Answers of the gym-goers

The majority (86.5%, n = 624) of the gym members assumed a beneficial effect of protein intake on hypertrophy or strength. More than the half (64.4%, n = 464) suspected an influence of the timing of protein intake and just below the half (45.9%, n = 331) believed that animal protein is superior to plant protein. High percentages assumed that creatine augments the effects of RT (73.8%, n = 532), carbohydrate intake acutely increases RT performance (74.5%, n = 537) and magnesium prevents cramps (82.9%, n = 598).

Clear majorities did not believe that RT would reduce flexibility (78.4%, n = 565) and that low-load, high volume RT would be as effective as high-load RT regarding maximum strength (68%, n = 490). However, about one half (48.7%, n = 351) claimed that low-load, high-volume RT would be as effective with regard to hypertrophy.

A large majority were convinced that volume-equated RT several times a week would be superior to RT once a week (90.3%, n = 651) and that RT over the full range of motion would be superior to partial range of motion (70.2%, n = 506). Conversely, less than half stated that RT to muscle failure would be required for hypertrophy and strength increases (48%, n = 346) and that RT with free weights would be superior to training on machines (45.2%, n = 326). Finally, two thirds (64.6%, n = 466) stated RT would be more effective in men vs. women.

### Correctness of classifications

The results of the χ^2^ goodness-of-fit tests are displayed in Table 2. In five of the 14 statements (truth: protein augments strength and hypertrophy, creatine augments strength, RT over full ROM is superior to RT for hypertrophy; myth: RT reduces flexibility, low-load, high-volume RT is as effective as high-load RT with regard to maximal strength) a majority selected the correct answer (p < 0.01). Similarly, in five statements (myth: timing of protein intake influences hypertrophy, carbohydrate intake acutely increases RT performance, magnesium prevents cramps, a higher RT frequency is superior to a lower frequency, RT has higher effects in men vs. women) the majority chose the incorrect answer (p < 0.01). For the remaining four statements (truth: low-load, high-volume RT is as effective as high-load RT with regard to hypertrophy; myth: animal protein is superior to plant protein, RT to muscle failure is necessary for hypertrophy, free weight RT is more effective than machine-based RT) there was no statistical difference between the number of correct and incorrect answers (p > 0.01).

**Table 2:** Myths and truths in RT as assumed by the total sample (n = 721) of gym-goers.

Statement	Truth in % (n)	Myth in % (n)	χ^2^ test (p-value)	Correct choice of majority (p < 0.01)
Protein supplementation augments strength and hypertrophy	**86.5 (624)**	*13.5 (97)*	χ2 = 385, p < 0.001*	**Yes**
Timing of protein intake influences hypertrophy	*64.4 (464)*	**35.6 (257)**	χ2 = 59.4, p < 0.001*	*No*
Animal protein affects hypertrophy more than plant protein	*45.9 (331)*	**54.1 (390)**	χ2 = 4.84, p = 0.028	*No*
Creatine augments strength	**73.8 (532)**	26.2 (189)	χ2 = 163, p < 0.001*	**Yes**
Carbohydrates increase performance in RT	*74.5 (537)*	**25.5 (184)**	χ2 = 173, p < 0.001*	*No*
Magnesium prevents cramps	*82.9 (598)*	**17.1 (123)**	χ2 = 313, p < 0.001*	*No*
RT reduces flexibility	*21.6 (156)*	**78.4 (565)**	χ2 = 232, p < 0.001*	**Yes**
Low-load RT is as effective as high-load RT with regard to hypertrophy	**48.7 (351)**	*51.3 (370)*	χ2 = 0.501, p = 0.479	*No*
Low-load RT is as effective as high-load RT with regard to maximal strength	*32 (231)*	**68 (490)**	χ2 = 93, p < 0.001*	**Yes**
Multiple RT is more effective than singular training	*90.3 (651)*	**9.7 (70)**	χ2 = 468, p < 0.001*	*No*
RT to muscle failure is necessary for hypertrophy	*48 (346)*	**52 (375)**	χ2 = 1.17, p = 0.28	*No*
RT over full ROM is superior to RT in partial ROM for hypertrophy	**70.2 (506)**	*29.8 (215)*	χ2 = 117, p < 0.001*	**Yes**
Men benefit from RT more than women	*64.6 (466)*	**35.4 (255)**	χ2 = 61.7, p < 0.001*	*No*
Free weight RT is more effective than machine-based RT	*45.2 (326)*	**54.8 (395)**	χ2 = 6.6, p = 0.01	*No*

Bold indicate the correct answer and italics reveal the incorrect answer based on the evidence of literature. Significant p- values are marked with asterisks.

The table lists absolute (n) and (%).

Sub-analyses for sex (men/ women) and age (young < 40 years, older > 39 years) are displayed in Tables 3 and 4. While men and women answered similarly in most cases (p > 0.01), χ^2^ goodness-of-fit tests identified significant differences for two statements (animal vs. plant protein, creatine supplementation, p < 0.01). The same applied to younger vs. older individuals who also answered differently in two cases (creatine supplementation, RT over full vs. partial range of motion, p < 0.01).

**Table 3:** Myths and truths in RT as assumed by women (n = 267) and men (n = 454).

Statement	Women (37%, n = 267)		Men (63%, n = 454)		χ^2^ test (p-value)
	Truth in % (n)	Myth in % (n)	Truth in %(n)	Myth in % (n)	
Protein supplementation augments strength and hypertrophy	84.64 (226)	*15.36 (41)*	87.22 (396)	*12.33 (56)*	χ^2^ = 1.32, p = 0.251
Timing of protein intake influences hypertrophy	*64.04 (171)*	35.96 (96)	*64.54 (293)*	35.46 (161)	χ^2^ = 0.017, p = 0.894
Animal protein affects hypertrophy more than plant protein	*32.58 (87)*	**67.42 (180)**	*53.74 (244)*	46.26 (210)	χ^2^ = 30.3, p < 0.001*
Creatine augments strength	65.54 (175)	*34.46 (92)*	**78.63 (357)**	*21.37 (97)*	χ^2^ = 14.9, p < 0.001*
Carbohydrates increase performance in RT	*70.04 (187)*	29.96 (80)	*77.09 (350)*	22.91 (104)	χ^2^ = 4.4, p = 0.034
Magnesium prevents cramps	*82.40 (220)*	17.60 (47)	*83.26 (378)*	16.74 (76)	χ^2^ = 0.088, p = 0.76
RT reduces flexibility	*20.22 (54)*	79.78 (213)	*22.47 (102)*	77.53 (352)	χ^2^ = 0.499, p = 0.48
Low-load RT is as effective as high-load RT with regard to hypertrophy	49.44 (132)	*50.56 (135)*	48.24 (219)	*51.76 (235)*	χ^2^ = 0.097, p = 0.756
Low-load RT is as effective as high-load RT with regard to maximal strength	*31.46 (84)*	68.54 (183)	*32.38 (147)*	67.62 (307)	χ^2^ = 0.065, p = 0.799
Multiple RT is more effective than singular training	*92.13 (246)*	7.87 (21)	*89.21 (405)*	10.79 (49)	χ^2^ = 1.64, p = 0.2
RT to muscle failure is necessary for hypertrophy	*44.19 (118)*	55.81 (149)	*50.22 (228)*	49.78 (226)	χ^2^ = 2.45, p = 0.118
RT over full ROM is superior to RT in partial ROM for hypertrophy	64.41 (172)	*35.58 (95)*	**73.57 (334**)	*26.43 (120)*	χ^2^ = 6.72, p = 0.010*
Men benefit from RT more than women	*66.29 (177)*	33.71 (90)	*63.66 (289)*	36.34 (165)	χ^2^ = 3.48, p = 0.06
Free weight RT is more effective than machine-based RT	*40.44 (108)*	59.55 (159)	*48.02 (218)*	51.98 (236)	χ^2^ = 0.129, p = 0.719

Underline indicate the correct answer and italics reveal the incorrect answer based on the evidence of literature. Significant p-values are marked with asterisks. In items with significant difference, bold values mark the group with the higher proportion answering correctly. The table lists absolute (n) and relative (%).

**Table 4:** Myths and truths in RT as assumed by young (< 40 years; n = 583) and older (> 39 years; n = 138) fitness member.

Statement	Young (80.8%, n = 583)		Old (19.2%, n = 138)		χ^2^ test (p-value)
	Truth in % (n)	Myth in % (n)	Truth in %(n)	Myth in % (n)	
Protein supplementation augments strength and hypertrophy	87.82 (512)	*12.17 (71)*	81.15 (112)	*18.84 (26)*	χ^2^ = 4.25; p = 0.039
Timing of protein intake influences hypertrophy	*62.95 (367)*	37.04 (216)	*70.28 (97)*	59.42 (41)	χ^2^ = 2.62, p = 0.105
Animal protein affects hypertrophy more than plant protein	*47.16 (275)*	52.83 (308)	*40.57 (56)*	59.42 (82)	χ^2^ = 1.95, p = 0.162
Creatine augments strength	**75.98 (443)**	*24.01 (140)*	64.49 (89)	*35.50 (49)*	χ^2^ = 7.62, p = 0.006*
Carbohydrates increase performance in RT	*75.98 (443)*	24.01 (140)	*68.11 (94)*	31.88 (44)	χ^2^ = 3.64, p = 0.057
Magnesium prevents cramps	*82.33 (480)*	17.66 (103)	*85.50 (118)*	14.49 (20)	χ^2^ = 0.795, p = 0.373
RT reduces flexibility	*20.92 (122)*	79.07 (461)	*24.63 (34)*	75.36 (104)	χ^2^ = 0.907, p = 0.341
Low-load RT is as effective as high-load RT with regard to hypertrophy	49.57 (289)	*50.42 (294)*	44.92 (62)	*55.07 (76)*	χ^2^ = 0.963, p = 0.236
Low-load RT is as effective as high-load RT with regard to maximal strength	*30.70 (179)*	69.29 (404)	*37.68 (52)*	62.31 (86)	χ^2^ = 2.5, p = 0.114
Multiple RT is more effective than singular training	*89.53 (522)*	10.46 (61)	*93.47 (129)*	6.52 (9)	χ^2^ = 1.98, p = 0.16
RT to muscle failure is necessary for hypertrophy	*49.91 (291)*	50.08 (292)	*39.85 (55)*	60.14 (83)	χ^2^ = 4.52, p = 0.033
RT over full ROM is superior to RT in partial ROM for hypertrophy	**72.38 (422)**	*27.61 (161)*	60.86 (84)	*39.13 (54)*	χ^2^ = 7.07, p = 0.008*
Men benefit from RT more than women	*65.69 (383)*	34.30 (200)	*60.144 (83)*	39.85 (55)	χ^2^ = 1.5, p = 0.22
Free weight RT is more effective than machine-based RT	*44.25 (258)*	55.74 (325)	*49.27 (68)*	50.72 (70)	χ^2^ = 1.14, p = 0.287

Underline indicate the correct answer and italics colours reveal the incorrect answer based on the evidence of literature. Significant p-values are marked with asterisks. In items with significant difference, bold values mark the group with the higher proportion answering correctly. The table lists absolute (n) and relative (%).

## Discussion

Examining the effects and mechanisms of exercise interventions is of paramount importance. However, while related research is published in high quantity, the actual translation of research findings into practice is less studied but equally relevant^76–80^. The present survey on popular claims in RT unveils a significant discrepancy between the available evidence and the awareness of Austrian gym-goers: In only five of 14 cases, a majority of individuals were able to correctly identify true or false claims on RT. Of note, in another five statements, a majority selected the incorrect answer. This means that the ability of participants to separate myths from truths was close to chance.

To the best of our knowledge, our study was the first to examine the evidence knowledge of gym members. This is noteworthy because RT is a particularly well-researched topic within exercise science, which is reflected by a bulk of available systematic reviews^5–7,20,36,81^. The finding of a poor transfer from science to practice is, however, in line with previous research demonstrating low awareness about scientific evidence in stretching^29^, exercise prescription^82^, and RT principles^83^. Contrarily to our survey, these earlier studies focused on sports and health professionals such as physiotherapists, teachers, or coaches. Considering that professionals represent decisive multipliers helping to bring research findings to the end users such as our participants, the low awareness in the gym members (which are end users) is not surprising. While the lack of knowledge in end users, among other factors, may be explained by a lack of awareness in the persons delivering exercise, it would be intriguing to gain insights into the reasons of the latter.

The recent growth of the sport and exercise industry has led to specialization and fragmentation of the individual scientific disciplines^84^. As a result, practitioners across health, fitness and sport are confronted with a multitude of specialized information about theoretical training foundations and changes in technology, making it difficult to keep up with the pace of knowledge generation^6,84^. Indeed, sports coaches have been shown to rely on articles from scientific journals only to a limited extent when aiming to find information for their daily practice. The most cited reason for this are time constraints^78,79,85–87^. In recent years, sourcing of high-quality research from scientific conferences, university courses or books has expanded to web-based resources, making these tools very popular^88^. Nevertheless, filtering out relevant information is becoming more and more difficult as misinformation on public health and sport science issues do exist on social media with a worrisome frequency^89–91^. Of note, potentially incorrect information on digital media does not only affect health and exercise professionals, but also end users from the general population.

Irrespective of the role of professionals’ knowledge generation, it could be assumed that novelty of information represents an important factor explaining awareness or non-awareness. It is estimated that only 14% of the leading scientific evidence for prevention and treatment in sports science and medicine become standard practice after an average of 17 years^80^. As a consequence, not only `how` but also `how fast` seems to be important when aiming to disseminate new knowledge. In our survey, older evidence (judging from the publication dates of the papers included in the identified systematic reviews), e.g., on the effects of protein supplementation, was indeed correctly identified by almost three quarters of the sample. However, the incorrect answers also contained older knowledge (e.g., timing of protein intake, prevention of cramps by means of magnesium). It can therefore be concluded that factors such as persons` interest in science, education, or science communication may rather explain the lack of awareness.

Besides revealing a generally low awareness of the evidence on RT, our survey found mostly similar response patterns in males vs. females as well as older vs. younger participants. However, there were some exceptions. Men (79%) more often than women (66%) correctly stated that creatine supplementation augments strength gains, which may be related to a higher interest of men in muscle mass and strength^92^. Contrarily, women (67% vs. 46%) more frequently than men identified the myth that animal protein affects hypertrophy more than plant protein. Possibly, the source of protein represents a more female-orientated topic, because the majority of vegetarians are women^93^. Regarding age, younger participants more often (81% vs 19%) knew that RT over the full ROM does not lead to reduced flexibility. While the reasons for this seem unclear, younger persons also more frequently (76% vs 65%) stated correctly that creatine augments strength and that RT over full ROM is superior to RT in partial ROM for hypertrophy (72% vs 61%). Besides a higher interest in strength, a reason maybe that training goals have been demonstrated to shift from performance towards health during the life course^92,94^.

Our results represent a call to action. They, once again, underscore that publication outcomes are subordinated to a poorly functioning system, resulting in a lack of ecological validity, a lack of translation into practice and ultimately a gap between research and practice^95^. Although various interventions in the field of RT have been tested in high-quality studies, summarized in reviews and meta-analyses, and recommended in guidelines, the translation to the end user remains a constant challenge^80^. Against this background, it is necessary to promote the understanding and application of evidence-based literature and to develop context-specific dissemination and implementation strategies for policy makers, experts, and the general public. Several strategies could help to better disseminate scientific knowledge. A broad approach seems advisable, distinguishing between communication to multipliers and end users. For both, information can be provided face-to face (e.g. in conferences, workshops etc.)^78^, or using indirect approaches such as brochures, flyers, advertisements, social media, podcasts, blogs, etc.^96,97^. With regard to the growing field of social media, sport science researchers tend to share their publications on blogs or web-based journals, social networking sites such as Facebook or Twitter, forums or bulletin boards^98^. The review of Bardus et al.^99^ recommends that researchers use popular social networking platforms to engage with the wider public, bringing their research closer to people, and better explaining their results. In addition, network platforms may be used to interact, discuss and disseminate research findings with like-minded people^77,97^. Furthermore, materials with data visualisations, infographics and information presented in a short, concise and easy-to-understand format for non-scientists can help the general population to better understand and accept new evidence^99^. The involvement of professional organisations, advisory boards, or working groups interested in the implementation of research findings can, furthermore, help incorporating the results into guidelines or policy measures^77,99,100^.

Finally, some methodological aspects merit consideration. We employed a census sampling method aimed at collecting data from all gyms in the largest cities of Carinthia, achieving an impressive response rate of 91%, which underscores the credibility of our data. However, it is important to note that more than half of the participants came from one city, Klagenfurt, which, although the largest city in the region, may lead to slight overrepresentation in our total sample.

We made a concerted effort to include participants of different ages and sexes, collecting data at various times throughout the day. The male majority (63%) in our study aligns with recent data from Germany^101^, indicating that a higher percentage of men (32%) are registered in fitness centers compared to women (26%). Additionally, our sample reflects the common trend that most individuals working out in gyms are young or middle-aged^101^ which also fits with our sample data.

While our census sampling approach, high response rate, and diverse participant composition suggest a high degree of representativeness, we must consider a potential selection bias. The gyms that participated may have attracted members with similar characteristics or beliefs, potentially influencing our findings. Furthermore, as participation was voluntary and not anonymous, the beliefs of those who declined to participate might differ from those who agreed, further impacting the generalizability of our results. Finally, as findings on RT are highly complex, some participants may have had troubles in understanding the questions and concepts asked herein. Yet, we aimed to counter this issue by using face validation and offering explanations and help during study participation.

## Conclusion

According to the present survey, Austrian gym goers display significant gaps in knowledge of RT methods and related practices. Our findings underline the importance of effective science communication and implementation research as the mere generation of knowledge does not mean that it is translated into behaviour in the general population.

## Supplementary Information

Supplementary Information 1.

Supplementary Information 2.

Supplementary Information 3.

## Data Availability

The datasets generated during and/or analysed during the current study are available from the corresponding author on reasonable request.
