# Supplementary material for: Knowledge of gym goers on myths and truths in resistance training
Source: Sci Rep. 2025 Jan 27;15:3401. doi: 10.1038/s41598-025-87485-8 (PMC11772780; doi:10.1038/s41598-025-87485-8)
Supplement: Supplementary file 1 — Supplementary Information 1. [file 41598_2025_87485_MOESM1_ESM.docx]

**Questionnaire**

Age in years: ___

Height in centimeters: ____

Weight in kilograms: ____

Sex (please choose one answer):

- Male

- Female

- Diverse

What is your primary training goal?

- Hypertrophy

- Strength

- General fitness

- Endurance

- Weight loss

- Others: _____

In which region is your gym?

Klagenfurt

Villach

Spittal an der Drau

Wolfsberg

Feldkirchen

Statements on resistance training

Protein supplementation augments the effects of RT with regard to hypertrophy and strength.

- True

- False

The timing of protein intake (supplementation or normal nutrition) influences the extent of RT-induced hypertrophy.

- True

- False

Animal protein is more effective than vegetable protein with regard to RT-induced gains in strength and hypertrophy.

- True

- False

Creatine supplementation augments the effects of RT with regard to strength.

- True

- False

Carbohydrate intake acutely increases RT performance.

- True

- False

Magnesium prevents cramps.

- True

- False

Regular strength training reduces flexibility.

- True

- False

Low-load, high-volume RT is as effective as high-load RT with regard to hypertrophy.

- True

- False

Low-load, high-volume RT is as effective as high-load RT with regard to maximal strength.

- True

- False

Volume-equated RT, performed several times a week, is more effective than RT performed once a week with regard to strength and hypertrophy.

- True

- False

Training to muscle failure is necessary to induce hypertrophy and strength increases.

- True

- False

RT over full ROM is superior to RT in partial ROM with regard to hypertrophy.

- True

- False

RT is more effective in men vs. women with regard to hypertrophy and strength.

- True

- False

RT with free weights is more effective than RT with machines with regard to strength and hypertrophy.

- True

- False
