# Supplementary material for: Knowledge of gym goers on myths and truths in resistance training
Source: Sci Rep. 2025 Jan 27;15:3401. doi: 10.1038/s41598-025-87485-8 (PMC11772780; doi:10.1038/s41598-025-87485-8)
Supplement: Supplementary file 2 — Supplementary Information 2. [file 41598_2025_87485_MOESM2_ESM.docx]

**Statements and evidence underpinning correct answers**

| **Statement** | **Correct answer** | **Notes** | **Evidence for correct answer** |
| --- | --- | --- | --- |
|  |  |  |  |
| Protein supplementation augments strength and hypertrophy. | truth | Protein supplementation supports muscle growth (hypertrophy) and strength, particularly when combined with resistance training. | ^31–38^ |
| Timing of protein intake influences hypertrophy. | myth | Protein timing (e.g., pre- or post-workout) is less important than total protein intake throughout the day for hypertrophy. | ^38–40^ |
| Animal protein affects hypertrophy more than plant protein. | myth | Both animal and plant proteins can stimulate hypertrophy if they provide adequate essential amino acids. | ^41,42^ |
| Creatine augments strength. | truth | Creatine is well-supported in literature for enhancing strength. | ^43–45^ |
| Carbohydrates increase performance in RT. | myth | Carbohydrates support endurance but are less impactful for maximal strength or hypertrophy in RT. | ^46,47^ |
| Magnesium prevents cramps. | myth | Evidence is inconclusive on magnesium preventing cramps in healthy adults. | ^48,49^. |
| RT reduces flexibility. | myth | Resistance training does not reduce flexibility and can even improve it when exercises involve full range of motion (ROM). | ^50,51^ |
| Low-load RT is as effective as high-load RT with regard to hypertrophy. | truth | Low-load RT can be as effective as high-load RT for hypertrophy, stimulating similar muscle growth, especially in untrained. | ^52–54,56–58^ |
| Low-load RT is as effective as high-load RT with regard to maximal strength. | myth | High-load resistance training is superior for maximizing strength due to greater motor unit recruitment. | ^52,55,59^ |
| Multiple RT is more effective than singular training. | myth | The frequency of resistance training does not have a significant or meaningful effect on muscle hypertrophy when the total training volume is matched. | ^60–64^ |
| RT to muscle failure is necessary for hypertrophy. | myth | Training to failure is not necessary for hypertrophy. Effective hypertrophy can be achieved with submaximal efforts and sufficient volume. | ^65–67^ |
| RT over full ROM is superior to RT in partial ROM for hypertrophy. | truth | Training through a full range of motion (ROM) recruits more muscle fibers and promotes better hypertrophy than partial ROM. | ^69–71^ |
| Men benefit from RT more than women. | myth | Both men and women can gain strength and hypertrophy from resistance training. | ^74,75^. |
| Free weight RT is more effective than machine-based RT. | myth | Both free weights and machines can be effective for resistance training. | ^72,73^ |

**References:**

31. Hartono, F. A., Martin-Arrowsmith, P. W., Peeters, W. M. & Churchward-Venne, T. A. The Effects of Dietary Protein Supplementation on Acute Changes in Muscle Protein Synthesis and Longer-Term Changes in Muscle Mass, Strength, and Aerobic Capacity in Response to Concurrent Resistance and Endurance Exercise in Healthy Adults: A Systematic Review. *Sports Med* **52**, 1295–1328 (2022).

32. Naclerio, F. & Larumbe-Zabala, E. Effects of Whey Protein Alone or as Part of a Multi-ingredient Formulation on Strength, Fat-Free Mass, or Lean Body Mass in Resistance-Trained Individuals: A Meta-analysis. *Sports Med* **46**, 125–137 (2016).

33. Nunes, E. A. *et al.* Systematic review and meta-analysis of protein intake to support muscle mass and function in healthy adults. *J Cachexia Sarcopenia Muscle* **13**, 795–810 (2022).

34. O’Bryan, K. R. *et al.* Do multi-ingredient protein supplements augment resistance training-induced gains in skeletal muscle mass and strength? A systematic review and meta-analysis of 35 trials. *Br J Sports Med* **54**, 573–581 (2020).

35. Pasiakos, S. M., McLellan, T. M. & Lieberman, H. R. The effects of protein supplements on muscle mass, strength, and aerobic and anaerobic power in healthy adults: a systematic review. *Sports Med* **45**, 111–131 (2015).

36. Morton, R. W. *et al.* A systematic review, meta-analysis and meta-regression of the effect of protein supplementation on resistance training-induced gains in muscle mass and strength in healthy adults. *Br J Sports Med* **52**, 376–384 (2018).

37. Tagawa, R. *et al.* Dose-response relationship between protein intake and muscle mass increase: a systematic review and meta-analysis of randomized controlled trials. *Nutr Rev* **79**, 66–75 (2020).

38. Wirth, J., Hillesheim, E. & Brennan, L. The Role of Protein Intake and its Timing on Body Composition and Muscle Function in Healthy Adults: A Systematic Review and Meta-Analysis of Randomized Controlled Trials. *J Nutr* **150**, 1443–1460 (2020).

39. Schoenfeld, B. J., Aragon, A. A. & Krieger, J. W. The effect of protein timing on muscle strength and hypertrophy: a meta-analysis. *Journal of the International Society of Sports Nutrition* **10**, 53 (2013).

40. Zhou, H.-H. *et al.* Effects of Timing and Types of Protein Supplementation on Improving Muscle Mass, Strength, and Physical Performance in Adults Undergoing Resistance Training: A Network Meta-Analysis. *Int J Sport Nutr Exerc Metab* **34**, 54–64 (2024).

41. Lim, M. T., Pan, B. J., Toh, D. W. K., Sutanto, C. N. & Kim, J. E. Animal Protein versus Plant Protein in Supporting Lean Mass and Muscle Strength: A Systematic Review and Meta-Analysis of Randomized Controlled Trials. *Nutrients* **13**, 661 (2021).

42. Messina, M., Lynch, H., Dickinson, J. M. & Reed, K. E. No Difference Between the Effects of Supplementing With Soy Protein Versus Animal Protein on Gains in Muscle Mass and Strength in Response to Resistance Exercise. *Int J Sport Nutr Exerc Metab* **28**, 674–685 (2018).

43. Burke, R. *et al.* The Effects of Creatine Supplementation Combined with Resistance Training on Regional Measures of Muscle Hypertrophy: A Systematic Review with Meta-Analysis. *Nutrients* **15**, 2116 (2023).

44. Delpino, F. M., Figueiredo, L. M., Forbes, S. C., Candow, D. G. & Santos, H. O. Influence of age, sex, and type of exercise on the efficacy of creatine supplementation on lean body mass: A systematic review and meta-analysis of randomized clinical trials. *Nutrition* **103–104**, 111791 (2022).

45. Imtiaz, D. *et al.* The Effect of Creatine Supplementation on Resistance Training-Based Changes to Body Composition: A Systematic Review and Meta-analysis. *Journal of strength and conditioning research* (2024) doi:10.1519/JSC.0000000000004862.

46. Henselmans, M., Bjørnsen, T., Hedderman, R. & Vårvik, F. T. The Effect of Carbohydrate Intake on Strength and Resistance Training Performance: A Systematic Review. *Nutrients* **14**, 856 (2022).

47. King, A., Helms, E., Zinn, C. & Jukic, I. The Ergogenic Effects of Acute Carbohydrate Feeding on Resistance Exercise Performance: A Systematic Review and Meta-analysis. *Sports Med* **52**, 2691–2712 (2022).

48. Garrison, S. R. *et al.* Magnesium for skeletal muscle cramps. *Cochrane Database Syst Rev* **9**, CD009402 (2020).

49. Sebo, P., Cerutti, B. & Haller, D. M. Effect of magnesium therapy on nocturnal leg cramps: a systematic review of randomized controlled trials with meta-analysis using simulations. *Fam Pract* **31**, 7–19 (2014).

50. Afonso, J. *et al.* Strength Training versus Stretching for Improving Range of Motion: A Systematic Review and Meta-Analysis. *Healthcare (Basel)* **9**, 427 (2021).

51. Alizadeh, S. *et al.* Resistance Training Induces Improvements in Range of Motion: A Systematic Review and Meta-Analysis. *Sports Med* **53**, 707–722 (2023).

52. Lacio, M. *et al.* Effects of Resistance Training Performed with Different Loads in Untrained and Trained Male Adult Individuals on Maximal Strength and Muscle Hypertrophy: A Systematic Review. *Int J Environ Res Public Health* **18**, 11237 (2021).

53. Carvalho, L. *et al.* Muscle hypertrophy and strength gains after resistance training with different volume-matched loads: a systematic review and meta-analysis. *Appl Physiol Nutr Metab* **47**, 357–368 (2022).

54. Currier, B. S. *et al.* Resistance training prescription for muscle strength and hypertrophy in healthy adults: a systematic review and Bayesian network meta-analysis. *Br J Sports Med* **57**, 1211–1220 (2023).

55. Schoenfeld, B. J., Wilson, J. M., Lowery, R. P. & Krieger, J. W. Muscular adaptations in low- versus high-load resistance training: A meta-analysis. *Eur J Sport Sci* **16**, 1–10 (2016).

56. Schoenfeld, B. J., Grgic, J., Ogborn, D. & Krieger, J. W. Strength and Hypertrophy Adaptations Between Low- vs. High-Load Resistance Training: A Systematic Review and Meta-analysis. *J Strength Cond Res* **31**, 3508–3523 (2017).

57. Lixandrão, M. E. *et al.* Magnitude of Muscle Strength and Mass Adaptations Between High-Load Resistance Training Versus Low-Load Resistance Training Associated with Blood-Flow Restriction: A Systematic Review and Meta-Analysis. *Sports Med* **48**, 361–378 (2018).

58. Lopez, P. *et al.* Resistance Training Load Effects on Muscle Hypertrophy and Strength Gain: Systematic Review and Network Meta-analysis. *Med Sci Sports Exerc* **53**, 1206–1216 (2021).

59. Refalo, M. C. *et al.* Influence of resistance training load on measures of skeletal muscle hypertrophy and improvements in maximal strength and neuromuscular task performance: A systematic review and meta-analysis. *J Sports Sci* **39**, 1723–1745 (2021).

60. Schoenfeld, B. J., Grgic, J. & Krieger, J. How many times per week should a muscle be trained to maximize muscle hypertrophy? A systematic review and meta-analysis of studies examining the effects of resistance training frequency. *Journal of Sports Sciences* **37**, 1286–1295 (2019).

61. Cuthbert, M. *et al.* Effects of Variations in Resistance Training Frequency on Strength Development in Well-Trained Populations and Implications for In-Season Athlete Training: A Systematic Review and Meta-analysis. *Sports Med* **51**, 1967–1982 (2021).

62. Grgic, J. *et al.* Effect of Resistance Training Frequency on Gains in Muscular Strength: A Systematic Review and Meta-Analysis. *Sports Med* **48**, 1207–1220 (2018).

63. Schoenfeld, B. J., Ogborn, D. & Krieger, J. W. Effects of Resistance Training Frequency on Measures of Muscle Hypertrophy: A Systematic Review and Meta-Analysis. *Sports Med* **46**, 1689–1697 (2016).

64. Grgic, J., Schoenfeld, B. J. & Latella, C. Resistance training frequency and skeletal muscle hypertrophy: A review of available evidence. *Journal of Science and Medicine in Sport* **22**, 361–370 (2019).

65. Davies, T., Orr, R., Halaki, M. & Hackett, D. Effect of Training Leading to Repetition Failure on Muscular Strength: A Systematic Review and Meta-Analysis. *Sports Med* **46**, 487–502 (2016).

66. Refalo, M. C., Helms, E. R., Trexler, E. T., Hamilton, D. L. & Fyfe, J. J. Influence of Resistance Training Proximity-to-Failure on Skeletal Muscle Hypertrophy: A Systematic Review with Meta-analysis. *Sports Med* **53**, 649–665 (2023).

67. Vieira, A. F. *et al.* Effects of Resistance Training Performed to Failure or Not to Failure on Muscle Strength, Hypertrophy, and Power Output: A Systematic Review With Meta-Analysis. *J Strength Cond Res* **35**, 1165–1175 (2021).

68. Kassiano, W. *et al.* Which ROMs Lead to Rome? A Systematic Review of the Effects of Range of Motion on Muscle Hypertrophy. *J Strength Cond Res* **37**, 1135–1144 (2023).

69. Pallarés, J. G. *et al.* Effects of range of motion on resistance training adaptations: A systematic review and meta-analysis. *Scand J Med Sci Sports* **31**, 1866–1881 (2021).

70. Schoenfeld, B. J. & Grgic, J. Effects of range of motion on muscle development during resistance training interventions: A systematic review. *SAGE Open Med* **8**, 2050312120901559 (2020).

71. Wolf, M., Androulakis-Korakakis, P., Fisher, J., Schoenfeld, B. & Steele, J. Partial Vs Full Range of Motion Resistance Training: A Systematic Review and Meta-Analysis. *International Journal of Strength and Conditioning* **3**, (2023).

72. Haugen, M. E. *et al.* Effect of free-weight vs. machine-based strength training on maximal strength, hypertrophy and jump performance - a systematic review and meta-analysis. *BMC Sports Sci Med Rehabil* **15**, 103 (2023).

73. Heidel, K. A., Novak, Z. J. & Dankel, S. J. Machines and free weight exercises: a systematic review and meta-analysis comparing changes in muscle size, strength, and power. *J Sports Med Phys Fitness* **62**, 1061–1070 (2022).

74. Refalo, M. C. *et al.* Biological Sex Differences in Absolute and Relative Changes in Muscle Size following Resistance Training in Healthy Adults: A Systematic Review with Meta-Analysis. Preprint at https://doi.org/10.51224/SRXIV.400 (2024).

75. Roberts, B. M., Nuckols, G. & Krieger, J. W. Sex Differences in Resistance Training: A Systematic Review and Meta-Analysis. *The Journal of Strength & Conditioning Research* **34**, 1448 (2020).
