# Supplementary material for: Knowledge of gym goers on myths and truths in resistance training
Source: Sci Rep. 2025 Jan 27;15:3401. doi: 10.1038/s41598-025-87485-8 (PMC11772780; doi:10.1038/s41598-025-87485-8)
Supplement: Supplementary file 3 — Supplementary Information 3. [file 41598_2025_87485_MOESM3_ESM.docx]

**Search Terms**

**Protein supplementation augments strength and hypertrophy.**
"resistance training" AND protein AND (supplementation OR ingestion OR intake) AND ("muscle mass" OR "lean body mass" OR “strength”) AND ("systematic review" OR "meta-analysis")

**Timing of protein intake influences hypertrophy.**
"resistance training" AND “protein” AND (supplementation OR ingestion OR intake) AND "timing" AND ("muscle mass" OR "lean body mass") AND ("systematic review" OR "meta-analysis")

**Animal protein affects hypertrophy more than plant protein.**
"resistance training" AND protein AND (supplementation OR ingestion OR intake) AND (animal OR plant OR "protein source") AND ("muscle mass" OR "lean body mass") AND ("systematic review" OR "meta-analysis")

**Creatine augments strength.**
"resistance training" AND creatine AND (supplementation OR ingestion OR intake) AND ("muscle mass" OR "lean body mass" OR “strength”) AND ("systematic review" OR "meta-analysis")

**Carbohydrates increase performance in RT.**
"resistance training" AND carbohydrates AND (supplementation OR ingestion OR intake) AND "performance" AND ("systematic review" OR "meta-analysis")

**Magnesium prevents cramps.**
"resistance training" AND magnesium AND (supplementation OR ingestion OR intake) AND ("muscle cramps” OR "cramps") AND ("systematic review" OR "meta-analysis")

**RT reduces flexibility.**
"resistance training" AND flexibility AND ("systematic review" OR "meta-analysis")

**Low-load RT is as effective as high-load RT with regard to hypertrophy.**
"resistance training" AND load AND ("muscle mass" OR "lean body mass" OR “hypertrophy“) AND ("systematic review" OR "meta-analysis")

**Low-load RT is as effective as high-load RT with regard to maximal strength.**
"resistance training" AND load AND ("maximal strength") AND ("systematic review" OR "meta-analysis")

**Multiple RT is more effective than singular training.**
("resistance training frequency” OR “training volume") AND ("muscle mass" OR "lean body mass") AND ("systematic review" OR "meta-analysis")

**RT to muscle failure is necessary for hypertrophy.**
"resistance training to muscle failure" AND ("muscle mass" OR "lean body mass" OR “hypertrophy”) AND ("systematic review" OR "meta-analysis")

**RT over full ROM is superior to RT in partial ROM for hypertrophy.**
"resistance training" AND (“ROM” OR “range of motion”) AND ("muscle mass" OR "lean body mass" OR “hypertrophy”) AND ("systematic review" OR "meta-analysis")

**Men benefit from RT more than women.**
"resistance training" AND (“sex” OR "gender differences" OR “men versus women”) AND ("muscle mass" OR "lean body mass") AND ("systematic review" OR "meta-analysis")

**Free weight RT is more effective than machine-based RT.**
"resistance training" AND (“free weight” OR “machine” OR "exercise modality") AND ("muscle mass" OR "lean body mass") AND ("systematic review" OR "meta-analysis")
